# Functional characterization of the water-soluble organic carbon of size-fractionated aerosol in the southern Mississippi Valley

**DOI:** 10.5194/acp-14-6075-2014

**Published:** 2014-06-20

**Authors:** M.-C. G. Chalbot, J. Brown, P. Chitranshi, G. Gamboa da Costa, E. D. Pollock, I. G. Kavouras

**Affiliations:** 1 University of Arkansas for Medical Sciences, Little Rock, Arkansas, USA; 2 National Center for Toxicological Research, Jefferson, Arkansas, USA; 3 University of Arkansas Stable Isotope Laboratory, Fayetteville, Arkansas, USA

## Abstract

The chemical content of water-soluble organic carbon (WSOC) as a function of particle size was characterized in Little Rock, Arkansas in winter and spring 2013. The objectives of this study were to (i) compare the functional characteristics of coarse, fine and ultrafine WSOC and (ii) reconcile the sources of WSOC for periods when carbonaceous aerosol was the most abundant particulate component. The WSOC accounted for 5 % of particle mass for particles with *δ*_p_ > 0.96 μm and 10 % of particle mass for particles with *δ*_p_ < 0.96 μm. Non-exchangeable aliphatic (H–C), unsaturated aliphatic (H–C–C=), oxygenated saturated aliphatic (H–C–O), acetalic (O–CH–O) and aromatic (Ar–H) protons were determined by proton nuclear magnetic resonance (^1^H-NMR). The total non-exchangeable organic hydrogen concentrations varied from 4.1 ± 0.1 nmol m^−3^ for particles with 1.5 < *δ*_p_ < 3.0 μm to 73.9 ± 12.3 nmol m^−3^ for particles with *δ*_p_ < 0.49 μm. The molar H/C ratios varied from 0.48 ± 0.05 to 0.92 ± 0.09, which were comparable to those observed for combustion-related organic aerosol. The R–H was the most abundant group, representing about 45 % of measured total non-exchangeable organic hydrogen concentrations, followed by H–C–O (27 %) and H–C–C= (26 %). Levoglucosan, amines, ammonium and methanesulfonate were identified in NMR fingerprints of fine particles. Sucrose, fructose, glucose, formate and acetate were associated with coarse particles. These qualitative differences of ^1^H-NMR profiles for different particle sizes indicated the possible contribution of biological aerosols and a mixture of aliphatic and oxygenated compounds from biomass burning and traffic exhausts. The concurrent presence of ammonium and amines also suggested the presence of ammonium/aminium nitrate and sulfate secondary aerosol. The size-dependent origin of WSOC was further corroborated by the increasing *δ*^13^C abundance from −26.81 ± 0.18 ‰ for the smallest particles to −25.93 ± 0.31 ‰ for the largest particles and the relative distribution of the functional groups as compared to those previously observed for marine, biomass burning and secondary organic aerosol. The latter also allowed for the differentiation of urban combustion-related aerosol and biological particles. The five types of organic hydrogen accounted for the majority of WSOC for particles with *δ*_p_ > 3.0 μm and *δ*_p_ < 0.96 μm.

## 1 Introduction

Atmospheric aerosols affect climate directly by absorption and scattering of incoming solar radiation and indirectly through their involvement in cloud microphysical processes (Pöschl, 2005; Ghan and Schwartz, 2007). They also influence atmospheric oxidative burden, visibility and human health (Sloane et al., 1991; Cho et al., 2005; Schlesinger et al., 2006). Organic carbon (OC) represents more than 40 % of aerosol mass in urban and continental areas, with the largest fraction of that being soluble in water, yet less than 20 % of that is chemically characterized (Putaud et al., 2004; Goldstein and Galbally, 2007). Moreover, the optical (absorption coefficient (*σ* (*λ*)), single scattering albedo (*ω_o_*)) and hydrophilic (vapor pressure ( $p_{o}^{L}$), evaporation, condensation and repartitioning) properties of organic aerosol cannot be described by any mathematical formulation of the properties of single compounds, since they are related to the number and type of chromophore (i.e., functional) groups and supra-molecular non-covalent interactions (e.g., hydrogen and van der Walls bonds) (Kavouras and Stephanou, 2002; Cappa et al., 2008; Rincon et al., 2009; Reid et al., 2011). Consequently, the incomplete characterization and the heterogeneity of organic aerosol limit our understanding of their fate and impacts.

OC is composed of primary and secondary compounds originating from anthropogenic and biogenic sources. The water-soluble fraction of organic carbon (WSOC) accounts for 30–90 % of OC, and it is composed of dicarboxylic acids, keto-carboxylic acids, aliphatic aldehydes and alcohols, saccharides, saccharide anhydrides, amines, amino acids, aromatic acids, phenols, organic nitrates and sulfates, and humic and fulvic acids (Timonen et al., 2008; Miyazaki et al., 2009; Pietrogrande et al., 2013; Wozniak et al., 2013). Proton nuclear magnetic resonance (^1^H-NMR) spectroscopy has been applied to characterize the WSOC content of urban, biogenic, marine, continental background and marine aerosol (Suzuki et al., 2001; Graham et al., 2002; Matta et al., 2003; Cavalli et al., 2004; Decesari et al., 2006, 2011; Finessi et al., 2012). Solid-state ^13^C cross polarized magic angle spinning (^13^C-CPMAS) NMR was also used to characterize atmospheric aerosol (Subbalakshmi et al., 2000; Sannigrahi et al., 2006). In addition, the secondary organic aerosol (SOA) composition was studied using two-dimensional (2-D) ^1^H–^1^H correlation spectroscopy (COSY) and ^1^H–^13^C heteronuclear single quantum coherence (HSQC) spectroscopy (Tagliavini et al., 2006; Maksymiuk et al., 2009). The analysis of the WSOC hydrophobic fraction by ^1^H and 2-D ^1^H–^1^H gradient COSY (gCOSY) NMR allowed for the detection of alkanoic acids based on resonances attributed to terminal methyl (CH_3_) at *δ*0.8 ppm, *n*-methylenes (*n*CH_2_) at *δ*1.3 ppm, and *α*-and *β*-methylenes (*α*CH_2_, *β*CH_2_) at *δ*2.2 ppm and *δ*1.6 ppm (Decesari et al., 2011). Carbohydrates and polyhydroxylated polynuclear aromatic hydrocarbons were identified on urban surface films in Toronto, Canada by ^1^H, 2-D ^1^H–^1^H total correlation spectroscopy (TOCSY) and semi-solid-state NMR (Simpson et al., 2006).

Cluster and positive matrix factorization (PMF) were applied to 21 ^1^H-NMR spectra using 200 (and 400) NMR bands as variables in Mace Head, Ireland (Decesari et al., 2011). Despite the inherent statistical errors associated with the use of a limited number of equations (samples, *n* = 21) to predict substantially more variables (*m* = 200 or *m* = 400), three to five factors were retained and assigned to methanesulfonate (MSA), amines, clean marine samples, polluted air masses and clean air masses. PMF was also applied to NMR and aerosol mass spectrometer data to apportion the sources of biogenic SOA in the boreal forest (Finessi et al., 2012). The four retained factors were attributed to glycols, humic-like compounds, amines + MSA and biogenic terpene-SOA originating from a polluted environment.

The overall aim of this study was to determine the compositional fingerprints of particulate WSOC for different particle sizes of urban aerosol in Little Rock, Arkansas. The specific objectives were to (i) compare the functional characteristics of coarse, fine and ultrafine WSOC and (ii) to reconcile the sources of WSOC by NMR spectroscopy and ^13^C isotope ratios. The Little Rock/North Little Rock metropolitan area is a mid-sized Midwestern urban area with PM_2.5_ (particles with diameter less than 2.5 μm) levels very close to the newly revised annual PM_2.5_ national ambient air quality standard of 12 μg m^−3^ (Chalbot et al., 2013a). OC was the predominant component, representing approximately ~ 55 % of PM_2.5_ mass, with the highest concentrations being measured during winter. The sources of fine atmospheric aerosol in the region included primary traffic particles, secondary nitrate and sulfate, biomass burning, diesel particles, aged/contaminated sea salt and mineral/road dust (Chalbot et al., 2013a). The region also experiences elevated counts of pollen in early spring due to the pollination of oak trees (Dhar et al., 2010). Due to the seasonal variation of weather patterns, the chemical content of aerosol may also be modified by regional transport of cold air masses from the Great Plains and Pacific Northwest in the winter (Chalbot et al., 2013a).

## 2 Materials and methods

### 2.1 Sampling

Seven-day urban size fractionated aerosol samples were collected every second week with a high-volume sampler in Little Rock, Arkansas in the winter and early spring of 2013 (February–March). The sampling duration was selected to reduce the effect of sampling biases (i.e., weekday/weekend or day/night) and obtain sufficient quantities for NMR analysis in each particle size range. The sampling site was located at the north end of the UAMS campus (34°45′3.69″ N and 92°19′10.28″ W). It was 20 m above the ground and approximately 100 m from West Markham Street with annual average daily traffic (AADT) of 13 000 vehicles. The I-630 Expressway is located 1 mile to the south of the sampling site (south end of the UAMS campus) with an AADT of 108 000 vehicles. The 6-lane (3 per direction) highway is an open below surface-level design to reduce air pollution and noise in the adjacent communities.

A five-stage (plus backup filter) Sierra Andersen Model 230 Impactor mounted on a high-volume pump was used (GMWL-2000, Tisch Environmental, Ohio, USA). Particles were separated into six size fractions on quartz fiber filters, according to their aerodynamic cutoff diameters at 50 % efficiency: (i) first stage: > 7.2 μm; (ii) second stage: 7.2–3.0 μm; (iii) third stage: 3.0–1.5 μm; (iv) fourth stage: 1.5–0.96 μm; (v) fifth stage: 0.96–0.5 μm; and (vi) backup filter: < 0.5 μm, at a nominal flow rate of 1.13 m^3^ min^−1^. We assumed an upper limit of 30 μm for the larger particles, in agreement with the specification for the effective cut point for standard high-volume samplers and to facilitate comparison with previous studies (Kavouras and Stephanou, 2002). After collection, filters were placed in glass tubes and stored in a freezer at −30 °C until extraction and analysis.

### 2.2 Materials

Quartz microfiber filters were purchased from Whatman (QM-A grade, 203 × 254 mm, Tisch Environmental, USA), were precombusted at 550 °C for 4 h and then kept in a dedicated clean glass container, with silica gel, to avoid humidity and contamination. Water (HPLC grade), deuterium oxide (NMR grade, 100 at. % D), 3-(trimethylsilyl)propionic acid-d4 sodium salt (98 at. % D), sodium phosphate buffer (for analysis, 99 %) and sodium azide (extra pure, 99 %) were purchased from Acros Organics (Fisher Scientific Company LLC, USA).

### 2.3 Analysis

A piece of the filters (1/10 of impactor stages (12.5 cm^2^) and 5.1 cm^2^ of the backup) was analyzed for *δ*^13^C by an elemental analyzer (NC2500 Carlo Erba, Milan Italy) interfaced via a Conflo III to a Delta Plus isotope ratio mass spectrometer (Thermo Finnigan, Bremen Germany) at the University of Arkansas Stable Isotope Laboratory. The samples were combusted at 1060 °C in a stream of helium with an aliquot of oxygen. Nitrogen oxides are reduced in a copper furnace at 600 °C. Resultant gases are separated using a 3 m chromatography column at 50 °C. Raw data are created using monitor gases, pure nitrogen and carbon dioxide. Raw results are normalized to the Vienna Pee Dee Belemnite (VPDB) using a combination of certified and in-house standards (Nelson, 2000). The relative isotope differences are expressed in permil versus VPDB calculated as follows:

(1) δC13=[R((C13/C12)sample)-R((C13/C12)standard)/R((C13/C12)sample)]×1000,

where *R*((^13^C/^12^C)_sample_) and *R*((^13^C/^12^C)_standard_) (VPDB) are the carbon isotope ratios of the sample and the standard, respectively (Coplen, 2011).

A 1 cm^2^ piece of each filter was extracted in 1 mL deionized water and an aliquot (20 μL) was analyzed for WSOC using a DRI Model 2001 Thermal/Optical thermal optical reflectance (TOR) carbon analyzer (Atmoslytic Inc., Calabasas, CA) following the Interagency Monitoring of PROtected Visual Environments (IMPROVE) thermal/optical reflectance (TOR) protocol at DRI’s Environmental Analysis Facility (Ho et al., 2006).

The remaining portion of each filter was extracted in 50 mL of ultrapure H_2_O for 1 h in an ultrasonic bath. The aqueous extract was filtered on a 0.45 μm polypropylene filter (Target2, Thermo Scientific), transferred into a pre-weighted vial (for the gravimetric determination of the total water-soluble extract, TWSE), dried using a SpeedVac apparatus and re-dissolved in 500 μL of deuterated water (D_2_O). A microbalance (Mettler-Toledo, model AB265-S) with a precision of 10 μg was used in a temperature-controlled environment. To minimize any variation in the pH of the samples and to block microbial activity, 100 μL of a buffer solution of disodium phosphate/monosodium phosphate (0.2 M Na_2_HPO_4_/0.2 M NaH_2_PO_4_, pH 7.4) and 100 μL of sodium azide (NaN_3_) (1 % *w*/*w*) were added into the sample, respectively. The ^1^H-NMR spectra were obtained on a Bruker Avance 500 MHz instrument equipped with a 5 mm double-resonance broad band (BBFO Plus Smart) probe at 298 K with 3600 scans, using spin lock, an acquisition time of 3.2 s, a relaxation delay of 1 s, and 1 Hz exponential line broadening and presaturation to the H_2_O resonance (Chalbot et al., 2013b). Spectra were apodized by multiplication, with an exponential decay corresponding to 1 Hz line broadening in the spectrum and a zero filling factor of 2. The baseline was manually corrected and integrated using the Advanced Chemistry Development NMR processor (Version 12.01 Academic Edition). The determination of chemical shifts (*δ*^1^H) was done relative to that of trimethylsilyl-propionic acid-d_4_ sodium salt (TSP-d_4_) (set at 0.0 ppm). The segment from *δ*4.5 ppm to *δ*5.0 ppm, corresponding to the water resonance, was removed from all NMR spectra. We applied the icoshift algorithm to align the NMR spectra (Savorani et al., 2010) and integrated the intensity of signals of individual peaks as well as in five ranges (Decesari et al., 2000, 2001; Suzuki et al., 2001). The saturated aliphatic region (H–C, *δ*0.6–*δ*1.8 ppm) was assumed to include protons from methyl, methylene and methine groups (R–CH_3_, R–CH_2_, and R–CH, respectively). The unsaturated aliphatic region (H–C–C=, *δ*1.8–*δ*3.2 ppm) contained signals of protons bound to aliphatic carbon atoms adjacent to a double bond, including allylic (H–C–C=C), carbonyl (H–C–C=O) or imino (H–C–C=N) groups. Secondary or tertiary amines (H–C–NR_2_) may also be present in the *δ*2.2–*δ*2.9 ppm region. The oxygenated saturated aliphatic region (H–C–O, *δ*3.2–*δ*4.4 ppm) contained alcohols, ethers and esters. The fourth region included acetalic protons (O–CH–O) with signals of the anomeric proton of carbohydrates and olefins (long-chain R–CH=CH–R, *δ*5.0–*δ*6.4 ppm). Finally, the fifth region (*δ*6.5–*δ*8.3 ppm) contained aromatic protons (Ar–H).

### 2.4 Calculations

The Lundgren diagrams and mass median aerodynamic diameter (MMAD) were used to describe the size distribution of particle mass, WSOC and non-exchangeable organic hydrogen concentrations ( $n_{\text{Conc}}^{o}$) as follows (Van Vaeck and Van Cauwenberghe, 1985; Kavouras and Stephanou, 2002):

(2) $$n_{\text{Conc}}^{o}=\frac{dC}{C_{t}\cdot \text{dlog}\left(d_{p}\right)},$$

where *C* is the concentration (μg m^−3^) for a given stage, *δ*_p_ is the aerodynamic diameter (μm), and *C*_t_ is the total concentration (μg m^−3^).

MMAD denotes the particle diameter (μm) with half of the particle mass, TWSE, WSOC or non-exchangeable organic hydrogen concentration above and the other half below. It was calculated stepwise as follows:

(3) $$(\int_{d_{1}}^{\text{MMAD}}C_{i}d(d_{p}))+\sum_{j=1}^{i-1}C_{j}=\frac{1}{2}C_{t}$$

where *δ*_1_ is the lower particle size (μm) for the *i*-impactor stage; *C_i_* and *C_j_* are the mass concentrations for the *i*- and *j* -impactor stages, respectively. If MMAD was higher than the upper particle size collected by the *i*-impactor stage, the calculation was repeated for the next stage. MMAD was calculated for the entire particle range, coarse particles (higher than 3.0 μm) and fine particles (less than 3.0 μm).

Multivariate linear regression analysis was used to attribute WSOC (in nmol m^−3^) to carbon associated with five types of non-exchangeable organic hydrogen as follows:

(4) $$\text{WSOC}=a_{1}\cdot {\left[H\right]}_{R-H}+a_{2}\cdot {\left[H\right]}_{H-C-C=}+a_{3}\cdot {\left[H\right]}_{O-C-H}+a_{4}\cdot {\left[H\right]}_{O-\text{CH}-O}+a_{5}\cdot {\left[H\right]}_{\text{Ar}-H}+a_{o},$$

where *α*_1_, *α*_2_, *α*_3_, *α*_4_ and *α*_5_ are the regression coefficients of non-exchangeable R–H, H–C–C=, O–C–H, O–CH–O and Ar–H concentrations (in nmol m^−3^). The intercept, *α*_0_, accounted for carbon not associated with the five organic hydrogen types such as carboxylic. The coefficient of variation of the root mean square error, CV(RMSE), was used to evaluate the residuals between measured and predicted WSOC values. It was defined as the RMSE normalized to the mean of the observed values:

(5) $$\text{CV}\;(\text{RMSE})=\frac{\text{RMSE}}{\bar{{\text{WSOC}}_{\text{measured}}}}=\frac{\sqrt{\sum_{i=1}^{n}{\left({\text{WSOC}}_{\text{predicted},i}-{\text{WSOC}}_{\text{measured},i}\right)}^{2}}}{n\cdot \bar{{\text{WSOC}}_{\text{measured}}}},$$

with RMSE being defined as the sample standard deviation of the differences between predicted values and observed values, n is the number of measurements and $\bar{{\text{WSOC}}_{\text{measured}}}$ is the average WSOC concentration.

## 3 Results and discussion

Table 1 shows the ambient temperature (°C), barometric pressure (torr), concentrations of major aerosol types and concentration diagnostic ratios of PM_2.5_ aerosol during the monitoring period at Little Rock at the nearest PM_2.5_ chemical speciation site (EPA AIRS ID: 051190007; Lat.: 34.756072°N; Long.: 92.281139°W) (Chalbot et al., 2013a). The site is located 3.6 km ENE (heading of 77.9°) of the UAMS campus. The Interagency Monitoring of PRO-tected Visibility Environments (IMPROVE) PM_2.5_ mass re-construction scheme was used to estimate the mass of the secondary inorganic (sulfate and nitrate) aerosol, organic mass, elemental carbon, soil dust and sea spray (Sisler, 2000).

Organic carbon (OC) was the predominant component of fine aerosol, accounting for 49 % of reconstructed PM_2.5_ mass, followed by secondary inorganic aerosol (40 %) and elemental carbon (EC) (7 %), which were comparable to those previously observed for the 2002–2010 period. The OC/EC ratio (4.58 ± 1.06) was comparable to those observed in the same region for the 2000–2010 period (Chalbot et al., 2013a) that identified biomass burning and traffic as the most important sources of carbonaceous aerosol in the region. This was further corroborated by the prevalence of soluble potassium, a tracer of biomass burning (K^+^/K ratio of 1.00 ± 0.28) (Zhang et al., 2013). The low K/Fe ratio (0.87 ± 0.25) and the ratios of mineral elements (Al, Si and Ca) were comparable to those previously observed in the US demonstrating the presence of soil dust (Kavouras et al., 2009; Chalbot et al., 2013a). The high molar ${\text{NH}}_{4}^{+}/{\text{SO}}_{4}^{2-}$ ratio suggested the complete neutralization of sulfate by ammonia, while the ${\text{SO}}_{4}^{2-}/S$ suggested the presence of other forms of S from oil and coal combustion.

### 3.1 Size distribution

The mean (± standard error) of particle mass, total water soluble extract (TWSE), WSOC and non-exchangeable organic hydrogen concentrations for the five regions (R–H, H–C–C=, H–C–O, O–CH–O and Ar–H) for each particle size range are presented in Table 2. In Table 2, the mean (± standard error) molar H/C ratio and *δ*^13^C for each particle size are also reported. The total particle mass concentration ranged from 1.6 ± 0.1 μg m^−3^ for particles with 0.96 < *δ*_p_ < 1.5 μm to 11.2 ± 2.8 μg m^−3^ for particles with *δ*_p_ < 0.49 μm. These levels were substantially lower than those measured in other urban areas but comparable to those observed in forests (Kavouras and Stephanou, 2002). The lowest and highest TWSE concentrations were 0.5 ± 0.1 μg m^−3^ and 5.4 ± 1.4 μg m^−3^, accounting for about 13 % of the largest (*δ*_p_ > 7.2 μm) and up to 61 % of the smallest particles (*δ*_p_ < 0.96 μm), respectively. The WSOC levels were 0.1 ± 0.1 μgC m^−3^ for particles with *δ*_p_ > 0.96 μm representing 10 % of TWSE and 5 % of particle mass and increased to 1.2 ± 0.1 μgC m^−3^ (22.2 % of TWSE and 10 % of particle mass) for particles with *δ*_p_ < 0.96 μm. The contribution of WSOC to particle mass was slightly higher than that computed in Hong Kong for PM_10_ particles, albeit at substantially lower levels (Ho et al., 2006). For comparison, the WSOC concentrations of size-fractionated aerosol collected during the dry season in the Amazon varied from 0.2 (3.5–10 μm) to 30.4 μgC m^−3^ (0.42–1.2 μm) (Tagliavini et al., 2006). The total non-exchangeable organic hydrogen concentrations varied from 4.1 ± 0.1 nmol m^−3^ for particles with 0.96 < *δ*_p_ < 1.5 μm to 73.9 ± 12.3 nmol m^−3^ for particles with *δ*_p_ < 0.49 μm, with R–H being the most abundant group, representing about 45 % of measured total non-exchangeable organic hydrogen concentrations followed by H–C–O (27 %) and H–C–C= (26 %).

The molar H/C ratio may provide information on the types of sources; however, it should be cautiously evaluated because of the inherent inability to identify exchangeable protons in hydroxyl, carboxylic and amine functional groups at neutral pH values by ^1^H-NMR (Duarte et al., 2007). H/C values higher than 2 were indicative of compounds with strong aliphatic components, while H/C values from 1 to 2 were typically associated with oxygenated or nitro-organic species, and H/C values lower than 1 suggested an aromatic signature (Fuzzi et al., 2001). The H/C molar ratios were 0.84 ± 0.02 and 0.92 ± 0.09 for particles with *δ*_p_ > 3.0 μm, decreased to 0.48 ± 0.05 for particles with 0.96 < *δ*_p_ < 3.0 μm and increased to 0.54 ± 0.05 and 0.73 ± 0.02 for smaller particles (*δ*_p_ < 0.96 μm). In a previous study, the molar H/C ratios for vegetation combustion and prescribed fire emissions collected very close to the fire front were 0.39 and 0.64–0.68, respectively, suggesting a strong polyaromatic content that was typically observed in combustion-related processes (Adler et al., 2011; Chalbot et al., 2013b).

**Figure 1:**
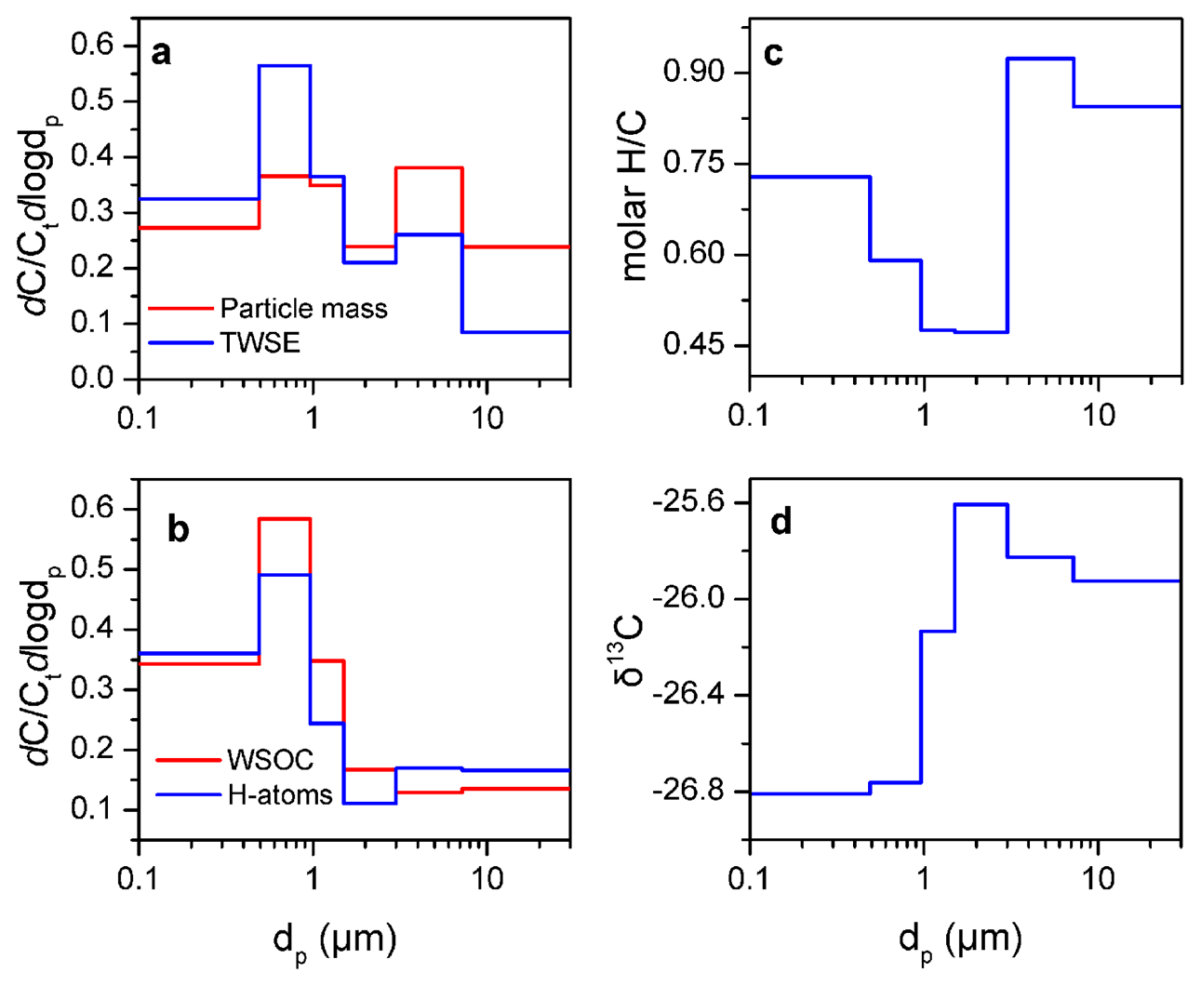
Size distribution for urban particle mass and TWSE **(a)**, WSOC and non-exchangeable organic hydrogen **(b)**, molar H / C ratio **(c)** and *δ*^13^C **(d)**.

The normalized concentration-based size distributions (i.e., Lundgren diagrams, Van Vaeck and Van Cauwenberghe, 1985) of particle mass, TWSE, WSOC and total non-exchangeable organic hydrogen concentrations are presented in Fig. 1a and b, respectively. Table 3 also shows the mass median aerodynamic diameter for each measured variable. Particle mass and TWSE followed a bimodal distribution with local maxima for particles with 0.49 < *δ*_p_ < 1.5 μm and 3.0 < *δ*_p_ < 7.2 μm. The first mode (i.e., fine particles) corresponded to MMADs of 0.39 ± 0.03 μm for particle mass and 0.39 ± 0.02 μm for TWSE, which was typical of those observed in other urban areas (Table 3) (Aceves and Grimalt, 1993; Kavouras and Stephanou, 2002). The MMADs of particle mass and TWSE for the second mode (i.e., coarse particles) were 9.15 ± 2.75 μm and 6.35 ± 0.45 μm, suggesting the presence of water-insoluble species (e.g., metal oxides) in larger particles (*δ*_p_ > 7.2 μm). The MMADs calculated for the whole range of particle sizes were 0.68 ± 0.48 μm and 0.46 ± 0.02 μm for particle mass and TWSE, respectively. This confirmed the accumulation of water-soluble species in the fine range. For WSOC and non-exchangeable organic hydrogen, the size distribution illustrated a one-mode pattern maximizing at particles with 0.49 < *δ*_p_ < 1.5 μm and corresponding to MMADs for the whole range of particle sizes of 0.43 ± 0.02 μm for WSOC and 0.41 ± 0.01 μm for non-exchangeable organic hydrogen. Coarse particles (> 3.0 μm) had an MMAD of 11.83 ± 2.20 μm for WSOC and 11.35 ± 1.45 μm, which was substantially higher than that computed for particle mass and TWSE, indicating the possible contribution of very large carbonaceous particles. Pollen particles from oak trees (*Quercus*) have diameters from 6.8 to 37 μm and only 10 % of them are present in smaller particles (0.8–3.1 μm) (Takahashi et al., 1995). The particle diameter of various types of tree and grass pollen ranged from 22 to 115 μm (Diehl et al., 2001). On the other hand, the fine particle MMADs for WSOC and non-exchangeable organic hydrogen of fine particles were 0.37 ± 0.01 μm and 0.34 ± 0.01 μm (comparable to those computed for particle mass and TWSE), indicating the considerable influence of WSOC on TWSE and particle mass in this size range.

### 3.2 Functional characterization

**Figure 2:**
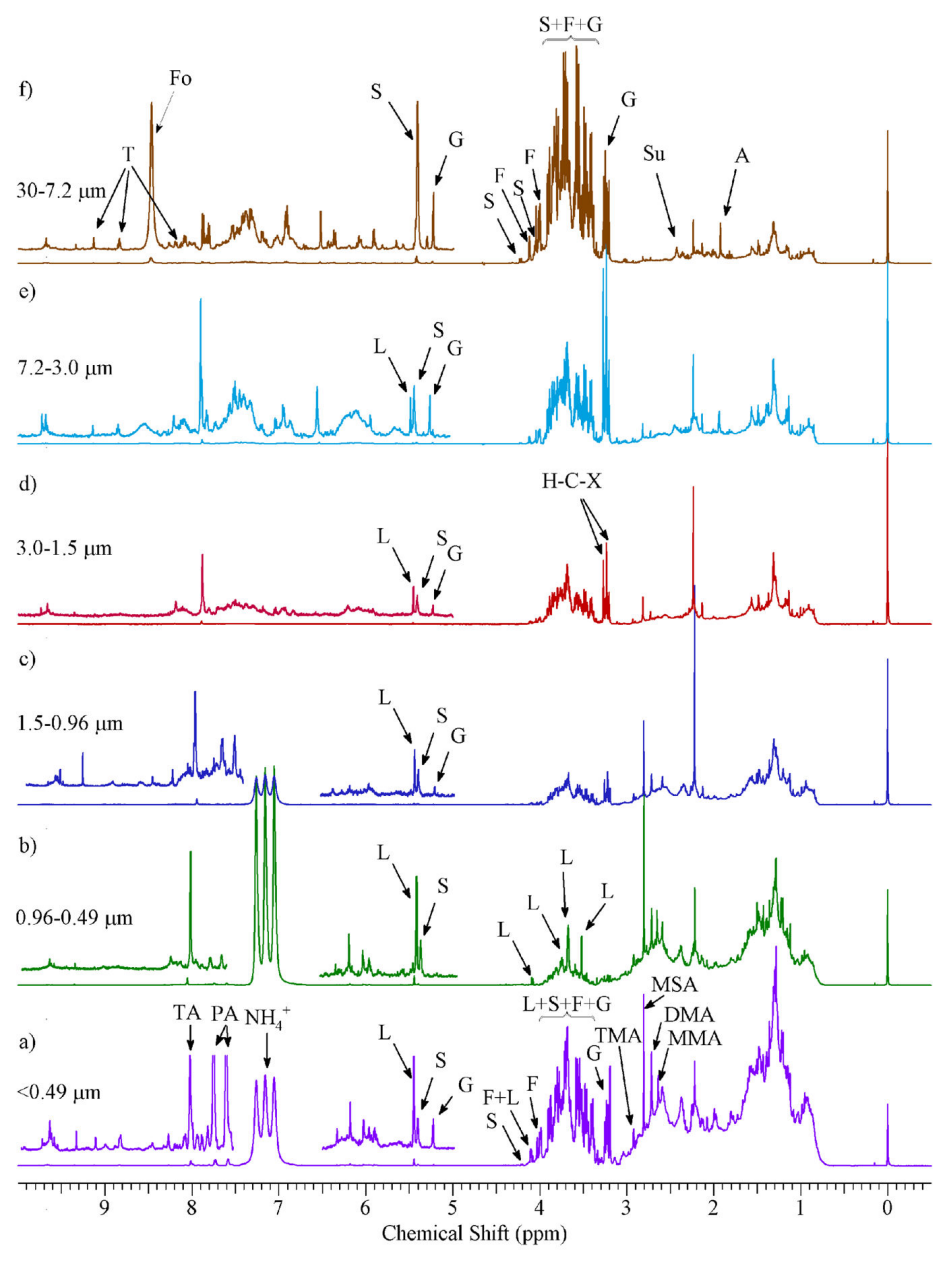
500 MHz 1H-NMR of size-fractionated WSOC. The segment from *δ*4.5 to *δ*5.0 ppm was removed from all NMR spectra due to H_2_O residues. The peaks were assigned to specific compounds as follows: formate (Fo), levoglucosan (L), glucose (G), sucrose (S), methanesulfonate (MSA), trimethylamine (TMA), succinate (Su), acetate (A), dimethylamine (DMA), monomethylamine (MMA), fructose (F), trigonelline (T), phthalic acid (PA), terephthalic acid (TA), ammonium ions ( ${\text{NH}}_{4}^{+}$).

**Figure 3:**
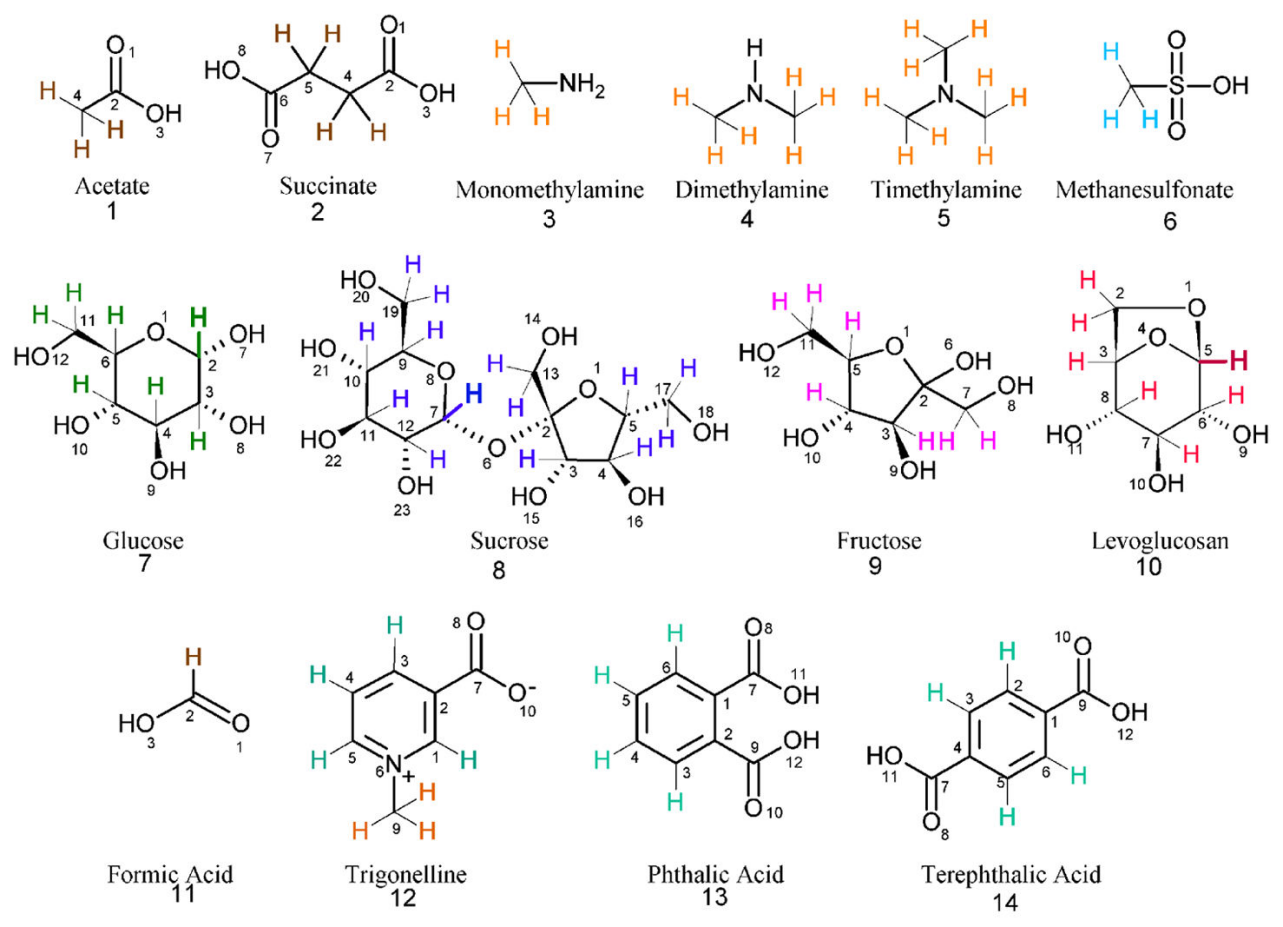
Structures of compounds assigned from the NMR spectra of fractionated aerosols. The protons responsible for the NMR signals are colored as follows: brown (bound to carbon alpha of the carboxylic acid group), orange (methyl groups bound to amines), light blue (bound to carbon alpha of the sulfonic acid group), green (glucose), blue (sucrose), purple (fructose), red (levoglucosan), light green (aromatic hydrogen). The H in bold indicates the signals in the 5.1–5.7 ppm range (see Fig. 4).

The ^1^H-NMR spectra of WSOC for different particle sizes are shown in Fig. 2. The structure of the compounds identified and the hydrogen assignment are shown in Fig. 3. The spectra are characterized by a combination of sharp resonances of the most abundant organic species and convoluted resonances of many organic compounds present at low concentrations. This section describes the variability of ^1^H-NMR spectra for different particles sizes in qualitative terms. A limited number of resonances were assigned to specific organic compounds using reference NMR spectra and in comparison with previous studies (Wishart et al., 2009).

The predominant peaks for particles with *δ*_p_ < 0.49 μm were those in the *δ*0.8 ppm to *δ*1.8 ppm range, with a somewhat bimodal distribution maximizing at *δ* ~ 0.9 ppm and *δ* ~ 1.3 ppm, respectively. They had previously been attributed to terminal methyl groups, alkylic protons and protons bound on C=O in compounds with a combination of functional groups and long aliphatic chains (Decesari et al., 2001). The ^1^H-NMR fingerprint in this region was comparable to that obtained for soil humic compounds, atmospheric humic-like species and urban traffic aerosol (Suzuki et al., 2001; Bartoszeck et al., 2008; Song et al., 2012; Chalbot et al., 2013b). It was previously observed that long chain (*C*_6_–*C*_30_) *n*-alkanoic acids, *n*-aldehydes and *n*-alkanes accumulated in particles with *δ*_p_ < 0.96 μm (Kavouras and Stephanou, 2002). The intensity of the convoluted resonances decreased for increasing particle sizes.

In the *δ*1.8–3.2 ppm range, the sharp resonances at *δ*1.92 ppm and *δ*2.41 ppm were previously assigned to aliphatic protons in *α* position in the COOH group in acetate (H-4 in Fig. 3) and in succinate (H-4 and H-5 in Fig. 3). These species were observed in the coarse fraction (Fig. 2e–f) but not in fine and ultrafine particles (Fig. 2a–c). These two acids (as well as formate) were typically associated with photo-oxidation processes and were present in the accumulation mode; however, Matsumoto et al. (1998) demonstrated that they were also present in sea spray coarse particles. Coarse acetate and formate were also observed in soil dust particles (Chalbot et al., 2013b).

The CH_3_ in mono-, di- and tri-methylamines (Fig. 3) was allocated to sharp resonances at *δ*2.59, *δ*2.72, and *δ*2.92 ppm, respectively. The major source of amines was animal husbandry and they were co-emitted with ammonia (Schade and Crutzen, 1995). They were present as vapors but they partition to aerosol phase by forming non-volatile aminium salts through scavenging by aqueous aerosol and reactions with acids, gas-phase acid–base reactions and displacement of ammonia from pre-existing salts (VandenBoer et al., 2011). The three amines were observed in particles with *δ*_p_ < 0.96 μm, which was consistent with previous studies and the suggested gas-to-particle partitioning mechanism (Mueller et al., 2009; Ge et al., 2011). Nitrate and sulfate particles constituted a considerable fraction of fine particles in Little Rock, Arkansas and it was associated with transport of air masses over the Great Plains and Upper Midwest, two regions with many animal husbandry facilities and the highest NH_3_ emissions in the US (Chalbot et al., 2013a). The presence of aminium/ammonium salts in the water-soluble fraction was also verified by the strong ammonium ^1^H–^14^N coupling signals at *δ*7.0–7.4 ppm (1 : 1 : 1 triplet, *J*_HN_ ~ 70 Hz) (Suzuki et al., 2001). Methanesulfonic acid (MSA) was also present (CH_3_ at *δ*2.81 ppm). MSA is a tracer of marine aerosols, formed from dimethylsulfide oxidation. We previously demonstrated the contribution of marine aerosols originating from the Gulf of Mexico in Little Rock (Chalbot et al., 2013a). MSA was accumulated to fine and ultrafine particles (*δ*_p_ < 1.5 μm) (Fig. 2d–f).

**Figure 4:**
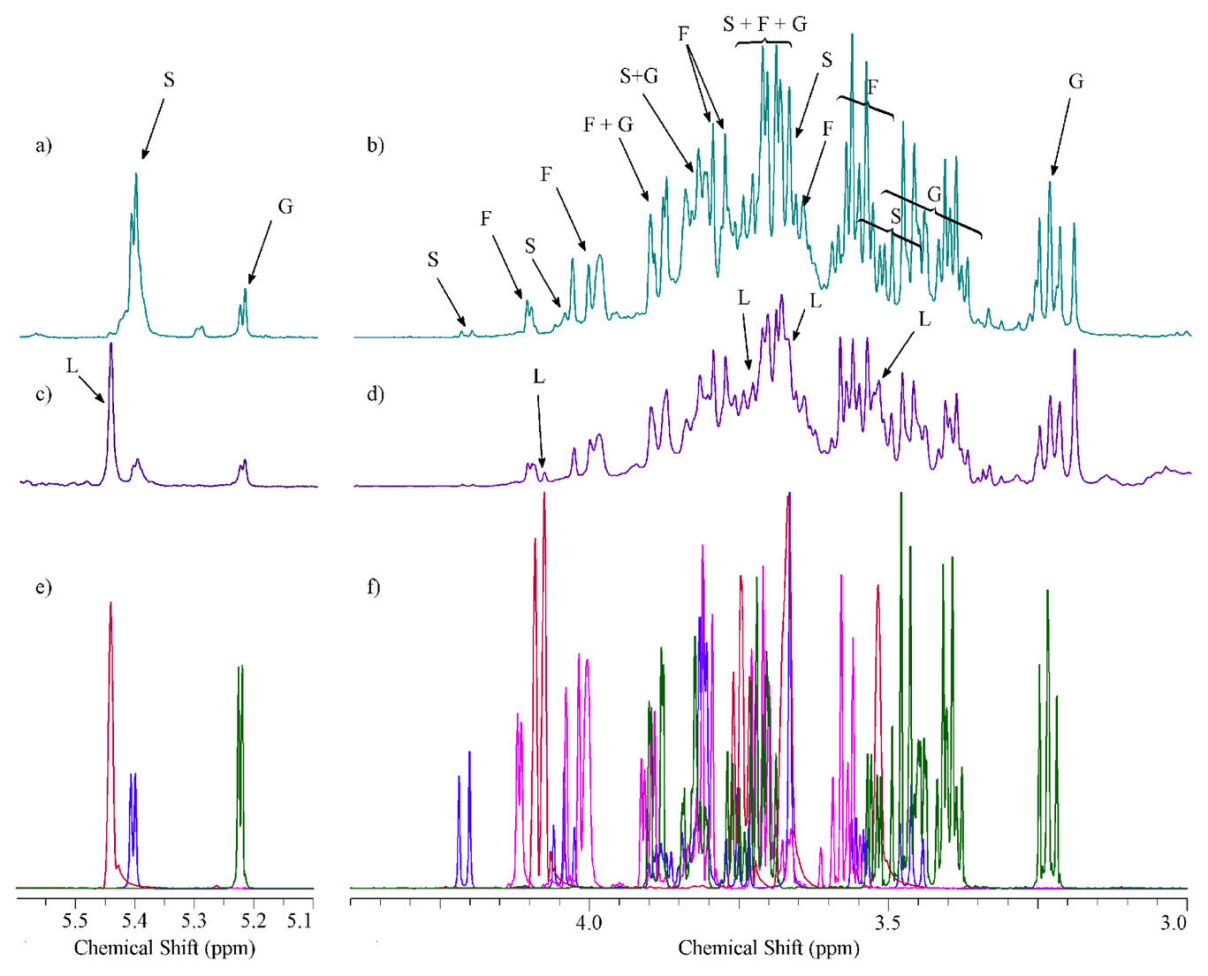
500 MHz *δ*3.0–4.4 ppm and *δ*5.1–5.6 ppm 1H-NMR segments for the largest **(a, b)** and smallest particles sizes **(c, d)** and reference NMR spectra **(e, f)** of levoglucosan (red), glucose (green), sucrose (blue) and fructose (purple).

Two segments of the carbohydrate region (*δ*3.0–4.4 ppm and *δ*5.1–5.6 ppm) of the ^1^H-NMR spectra for the largest and smallest particles sizes are presented in Fig. 4a–d, respectively. In addition, Fig. 4e and f show the combination of individual NMR reference spectra for glucose (HMDB00122), sucrose (HMDB00258), fructose (HMDB00660) and levoglucosan (HMDB00640) retrieved from the Human Metabolome Database (HMDB) NMR databases (Wishart et al., 2009). The ^1^H-NMR spectra of size-fractionated WSOC contain both convoluted resonances illustrated by a broad envelope in the spectra, and sharp resonances. For particles with *δ*_p_ > 7.2 μm, the spectra were dominated by sharp resonances assigned to glucose (G in Fig. 2; H-3, multiplet at *δ*3.24 ppm; H-5, multiplet at *δ*3.37–3.43 ppm; H-6, multiplet at *δ* 3.44–3.49 ppm; H-3, multiplet at *δ*3.52 ppm; H-4, multiplet at *δ*3.68–3.73 ppm; H-11, multiplet at *δ*3.74–3.77 ppm and 3.88–3.91 ppm; H-6 and H-11, multiplet at 3.81–3.85 ppm; and alpha H-2, doublet at 5.23 ppm), sucrose (S in Fig. 2; H-10, multiplet at 3.46 ppm; H-12, multiplet at 3.55 ppm; H-13, singlet at *δ*3.67 ppm; H-11, multiplet at 3.75 ppm; H-17 and H-19, multiplet at *δ*3.82 ppm; H-9, multiplet at 3.87 ppm; H-5, multiplet at 3.89 ppm; H-4, multiplet at *δ*4.06 ppm; H-3, doublet at *δ*4.22 ppm and H-7, doublet at 5.41 ppm) and fructose (F in Fig. 2; H-7, multiplet at *δ*3.55–3.61 ppm; H-7 and H-11, multiplet at *δ*3.66–3.73 ppm; H-3, H-5 and H-11, multiplet at *δ*3.79–3.84 ppm; H-4, multiplet at *δ*3.89–3.91 ppm; H-5 and H-11, multiplet at *δ*3.99–4.04 ppm; H-3 and H-4, multiplet at *δ*4.11–4.12 ppm). The overall NMR profile in this range was comparable to that observed for the combination of glucose, sucrose and fructose reference spectra (Fig. 4e and f) and atmospheric pollen (Chalbot et al., 2013c). The intensity of proton resonances in the *δ*3.30–4.15 ppm range was highest for the largest (*δ*_p_ > 7.2 μm) and smallest (*δ*_p_ < 0.49 μm) particles and decreased approximately eight times for particles in the 0.96 < *δ*_p_ < 1.5 μm size range (Fig. 2a–f). Carbohydrates of biological origin (i.e., pollen) were typically associated with large particles; however, they were also observed in fine biomass burning or biogenic aerosols (Bugni and Ireland, 2004; Medeiros et al., 2006; Agarwal et al., 2010; Fu et al., 2012; Chalbot et al., 2013c). The diameter of airborne fragments of fungal and pathogenic material may be < 1 μm, with their highest concentrations being measured in fall and spring (Yamamoto et al., 2012). The presence of sugars in particles with *δ*_p_ < 0.49 μm may be due to particle breakup during sampling, an inherent artifact of impaction (Kavouras and Koutrakis, 2001). It has been shown that this error may account for up to 5 % of the particle mass for particles with diameters higher than the cut-off point of the impactor stage. In our study, this would add up to 0.05 nmol m^−3^ (or 0.2 %) of the non-exchangeable H–C–O concentration to the concentration of particles with *δ*_p_ < 0.49 μm, suggesting the negligible influence of sampling artifacts on the observed size distribution.

Levoglucosan (H-6, multiplet at *δ*3.52 ppm; H-7 and H-8, multiplet at *δ*3.67; H-2, multiplet at *δ*3.73–3.75 ppm and at 4.08 ppm; H-5, singlet at 5.45 ppm (H-3 at 4.64 ppm; this peak was not visible due to interferences from solvent residues)) was also detected in the carbohydrate region of the ultrafine and fine ^1^H-NMR. Its concentrations, computed using the resonance at *δ*5.45 ppm, ranged from 1.1 ng m^−3^ for particles with *δ*_p_ > 7.2 μm to 19.1 ng m^−3^ for particles with 0.49 < *δ*_p_ < 0.96 μm. The mean total concentration was 33.1 ng m^−3^, which was comparable to those observed in US urban areas (Hasheminassab et al., 2013). Levoglucosan was previously observed in the ^1^H-NMR spectra of aerosol samples dominated by biomass burning in the Amazon (Graham et al., 2002).

A group of very sharp resonances between *δ*3.23 and *δ*3.27 ppm was observed with increasing intensity as particle size increased (Fig. 2a–f). These peaks were previously attributed to H–C–X (where X=Br, Cl, or I) functional groups (Cavalli et al., 2004).

The intensities of proton resonances in the aromatic region were very low, accounting for 0.3 to 1.2 % of the total non-exchangeable hydrogen concentration, which was consistent with those observed in other studies (Decesari et al., 2007; Cleveland et al., 2012). Resonances were previously attributed to aromatic amino acids and lignin-derived structures, mainly phenyl rings substituted with alcohols OH, methoxy groups O–CH_3_ and unsaturated C=C bonds, and their combustion products (Duarte et al., 2008). Four organic compounds were identified by means of their NMR reference spectra. These were formate (Fo in Fig. 2; H-2, singlet at 8.47 ppm), trigonelline (T in Fig. 2; H-4, multiplet at *δ*8.09 ppm; H-5 and H-3, multiplet at *δ*8.84 ppm; H-1, singlet at *δ*9.13 ppm; H-9, singlet at 4.42 ppm), phthalic acid (P in Fig. 2; H-4 and H-5, multiplet at *δ*7.58 ppm; H-3 and H-6, multiplet at *δ*7.73 ppm) and terephthalic acid (TA in Fig. 2; H-6, H-2, H-5 and H-3, multiplet at *δ*8.01). Formate and trigonelline were only observed in particles with *δ*_p_ > 7.2 μm due to the absorption of formate on pre-existing particles and the biological origin of trigonelline (Chalbot et al., 2013c). The phthalic acid and its isomer, terephthalic acid, were only observed in particles with *δ*_p_ < 0.49 μm. These compounds have already been detected in urban areas and vehicular exhausts (Kawamura and Kaplan, 1987; Alier et al., 2013). They may also be formed during the oxidation of aromatic hydrocarbons, but oxidation reactions are not favored by prevailing atmospheric conditions in the winter in the study area (Kawamura and Yasui, 2005).

Overall, the qualitative analysis of 1H-NMR spectra showed the prevalence of sugars in larger particles and a mixture of aliphatic and oxygenated compounds associated with combustion-related sources such as biomass burning and traffic exhausts. The presence of ammonium/aminium salts, probably associated with nitrate and sulfate secondary aerosol, was also identified.

### 3.3 Source reconciliation

**Figure 5:**
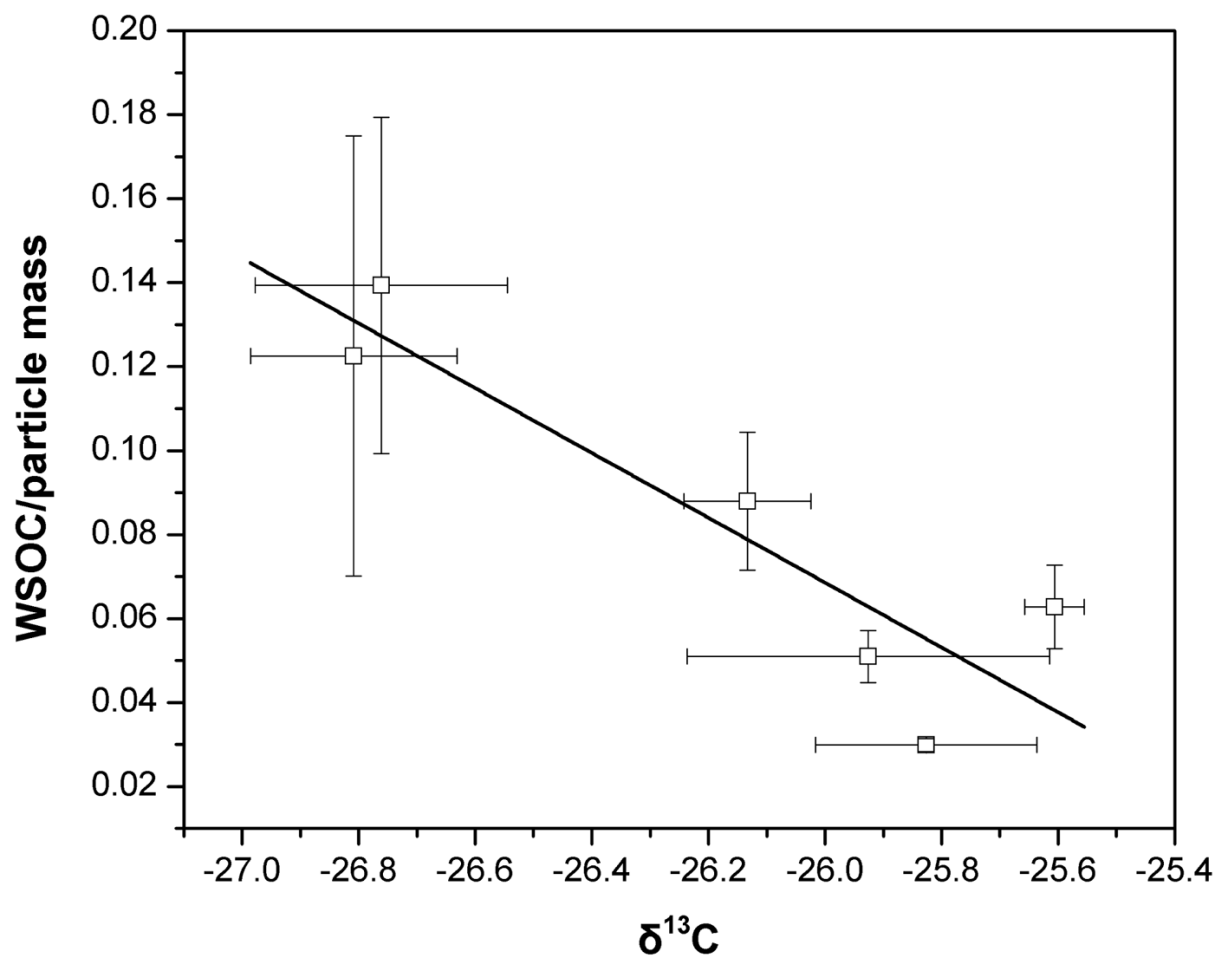
Association of the 13C isotopic ratio with the WSOC / particle mass ratio.

The *δ*^13^C ratios and the relative presence of the different types of protons were further analyzed to identify the sources of WSOC. Stable ^13^C isotope ratios have been estimated for different types of organic aerosol. The compounds associated with marine aerosols emitted via sea spray have *δ*^13^C values from −20 to −22 ‰ (Fontugne and Duplessy, 1981), and a decrease in the *δ*^13^C to −26 ± 2 ‰ of marine tropospheric aerosols has been associated with the presence of continental organic matter (Cachier et al., 1986; Chesselet et al., 1981). The carbon isotopic ratio of particles from the epicuticular waxes of terrestrial plants is related to the plant physiology and carbon fixation pathways, with *C*_3_ plants being less enriched in ^13^C (from −20 ‰ to −32 ‰) than the *C*_4_ plants (−9 to −17 ‰) (Collister et al., 1994; Ballantine, 1998). The *δ*^13^C ratios of organic aerosol from combustion of unleaded gasoline and diesel are −24.2 ± 0.6 ‰ and −26.2 ± 0.5 ‰, respectively (Widory et al., 2004). Atmospheric aging during transport increases the isotopic ratios (Aggarwal et al., 2013). In our study, the *δ*^13^C values increased from −26.81 ± 0.18 ‰ for the smallest particles (*δ*_p_ < 0.49 μm) to −25.93 ± 0.31 ‰ for the largest particles (*δ*_p_ > 7.2 μm), indicating a size-dependent mixture of anthropogenic and biogenic sources. Figure 5 shows the association (*r*_2_ = 0.69) between the WSOC-to-particle mass ratio and *δ*^13^C for particles with different sizes. The ^13^C enrichment of WSOC for low WSOC-to-particle mass ratios indicated the negligible effect of atmospheric aging. The predominance of R–H, moderate H / C ratios and low *δ*^13^C for the smaller particles (*δ*_p_ < 0.96 μm) were consistent with the contribution of combustion-related sources (Fig. 1c and d). A high *δ*^13^C ratio, the prevalence of oxygenated groups (H–C–O) and a high H / C ratio such as those observed for coarse particles (*δ*_p_ > 3.0 μm) would point towards aged organic aerosol; however, the large size of particles with these characteristics and the low WSOC-to-particle mass ratio suggested the influence of primary biogenic particles (Table 1).

**Figure 6:**
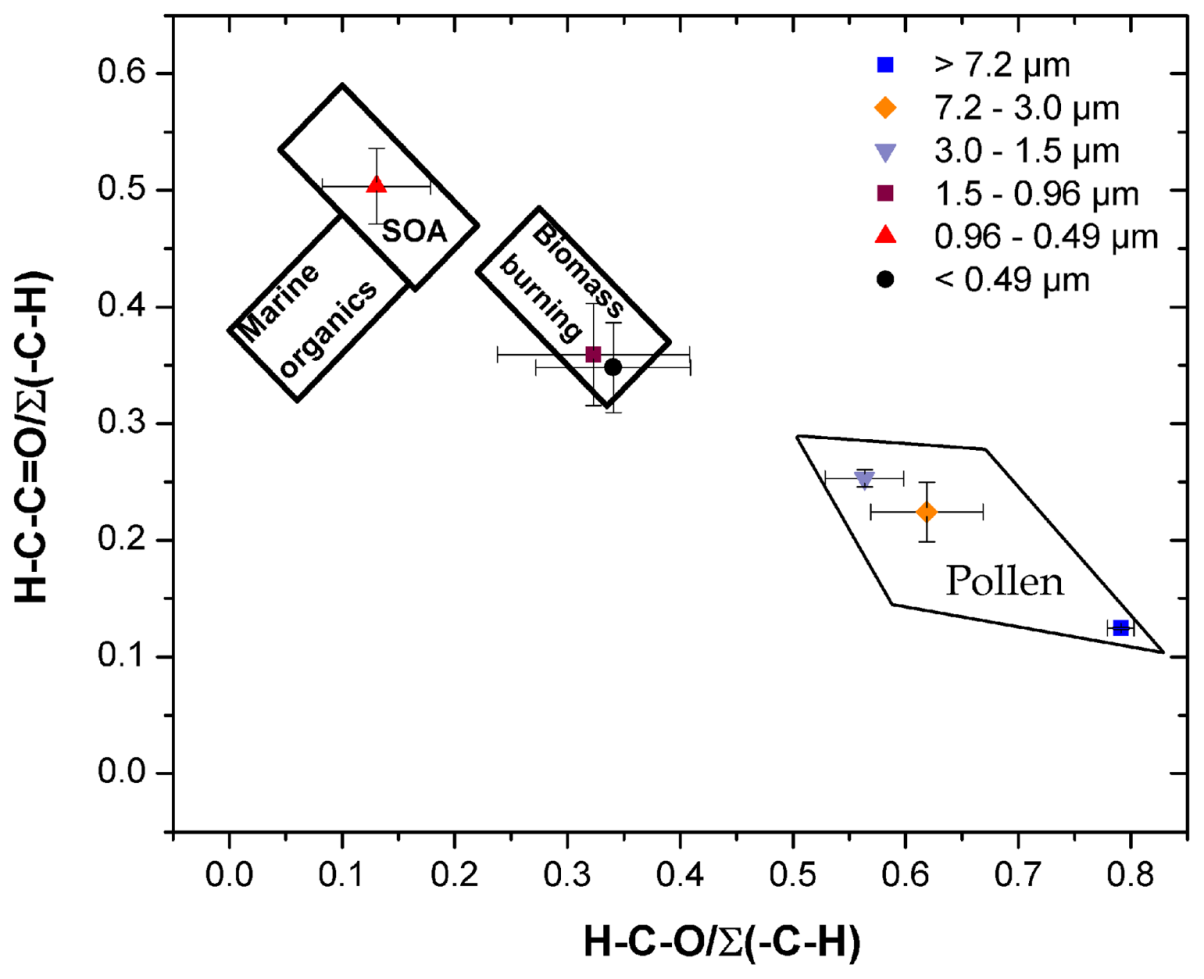
Functional group distributions of WSOC for each impactor stage. The boundaries of biomass burning, marine and secondary organic aerosol were obtained from Decesari et al. (2007).

By plotting the ratios of calculated carboxylics and ketones (H–C–C=O) (by subtraction of the Ar–H from the H–C–C= region) to the total aliphatics (Σ (H–C–)) and H–C–O / Σ (H–C–), Decesari et al. (2007) assigned three areas of the plot to OC sources, namely, biomass burning, marine and secondary organic aerosol. The Σ (H–C–) included the saturated (H–C–O, hydroxyls) and the unsaturated oxygenated (HC–C=O in acids and ketones) groups, the benzylic (H–C–Ar) groups, the unfunctionalized alkyl (H–C) groups, and minor contributions from other aliphatic groups such as the sulfonic group of MSA. More recently, Cleveland et al. (2012) demonstrated the need to define the boundaries for urban and industrial aerosol that were described by moderate H–C–O / Σ (H–C–) and H–C–C=O / Σ (H–C) ratios. Figure 6 depicts the locations of the urban size-fractionated samples collected in this study, in relation to the three aforementioned WSOC sources. Overall, the H–C–C=O / Σ (H–C–) ratio increased and the H–C–O / Σ (H–C–) ratio decreased for decreasing particle sizes. The H–C–C=O / Σ (H–C–) varied from 0.12 to 0.50 and the H–CO / Σ (H–C–) varied from 0.13 to 0.79. The data points for the smaller particles (*δ*_p_ < 1.5 μm) were within the boundaries of biomass burning and SOA, demonstrating the significance of wood burning emissions. The presence of biological aerosol with *δ*_p_ > 3.0 μm yielded low H–C–C=O / Σ (H–C–) ratios with a clear separation from combustion-related processes. These findings, in conjunction with those presented by Decesari et al. (2007) and Cleveland et al. (2012), suggest distinct signatures for different sources of organic aerosol that, once defined, may be used to determine the predominant sources of particulate WSOC.

The MMAD for the specific types of organic hydrogen may also provide qualitative information on the origin of organic aerosol. The MMAD of an organic species is found at a significantly smaller particle size than for the total aerosol when condensation (i.e., hot vapors cooling) or a gas-to-particle conversion mechanism prevails. The MMAD for R–H and H–C–C= were comparable, indicating a common origin. Their MMAD values for the total particle size range, coarse particles and fine particles were lower than those computed for particle mass and WSOC that can be interpreted by the condensation of hot vapor emissions from fossil fuel combustion and wood burning. This was further corroborated by the similar MMAD values for the total particle size range and fine particles for R–H and H–C–C=.

However, different trends were observed for O–C–H, O–CH–O and Ar–H. For O–C–H, the MMADs suggested a dual origin: (i) a strong condensation pathway for fine particles with an MMAD value (0.31 ± 0.01 μm) for fine particles that was lower than that for the entire particle size range (0.48 ± 0.02 μm) and fine MMADs for particle mass and WSOC, and (ii) a dominant primary (i.e., direct particle emissions) pathway for coarse particles with the highest MMAD values for all particle metrics in this study (13.05 ± 1.95 μm). Lastly, the high MMAD values for O–CH–O and Ar–H for the entire and fine particle size ranges as compared to those computed for the other types of organic hydrogen, particle mass and WSOC pointed towards emissions of primary particles.

### 3.4 WSOC reconstruction

**Figure 7:**
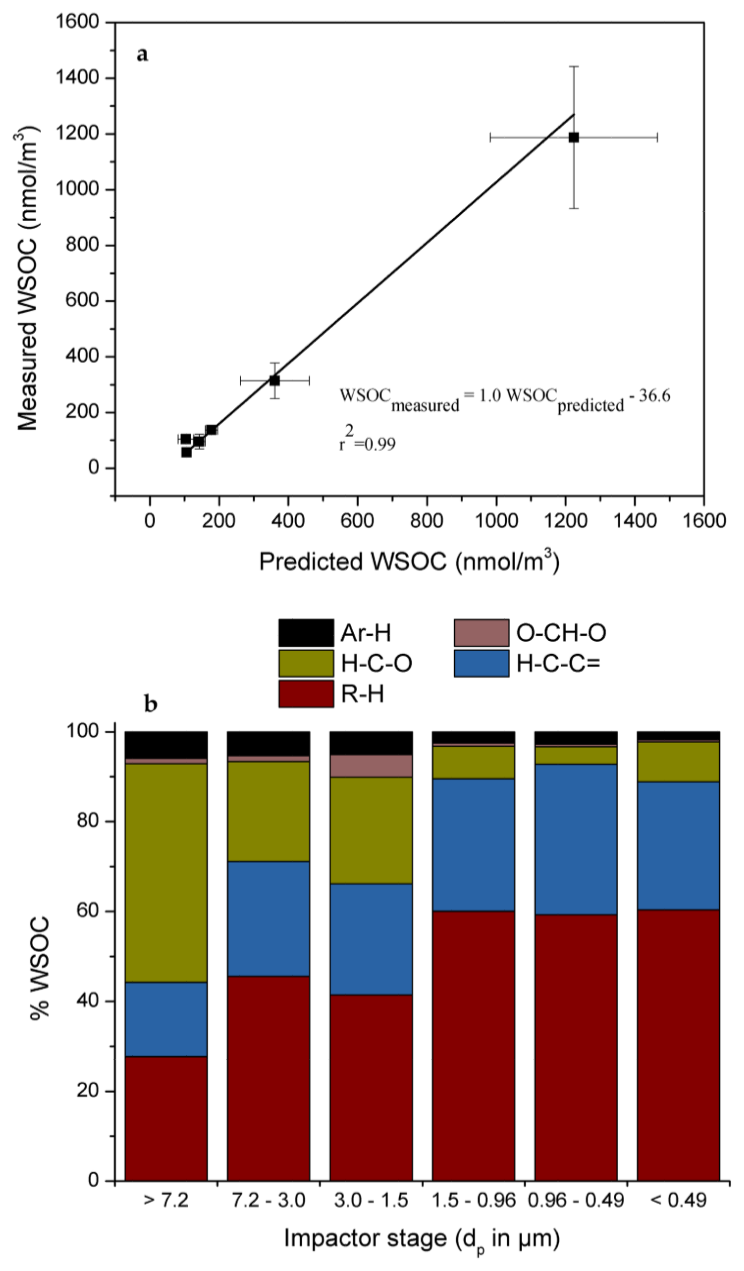
Measured and predicted WSOC concentrations **(a)** and contributions of R–H, H–C–C=, H–C–O, O–CH–O and Ar–H on WSOC **(b)** for each impactor stage of urban aerosol.

In this section, we estimated the contribution of each type of non-exchangeable organic hydrogen to WSOC levels by regression analysis (Eq. 4) without making any assumptions about the H / C ratio. The regression coefficients are estimates of the product of the H / C ratio and the relative presence of the functional group in the overall organic composition. Figure 7a presents a comparison between the measured and calculated WSOC levels and Fig. 7b illustrates the attribution of WSOC concentrations to specific types of carbon using the same definitions as for the non-exchangeable protons, i.e., saturated aliphatic (R–H), unsaturated aliphatic (H–C–C=), oxygenated saturated aliphatic (H–C–O), acetalic (O–CH–O) and aromatic (Ar–H), respectively. There was very good agreement (*r*_2_ = 0.99, slope of 0.9964) between measured WSOC and predicted WSOC concentrations with an CV(RMSE) of 0.02 (or 2 %). The R–H carbon was the predominant type of WSOC for particles with *δ*_p_ < 7.2 μm (41–60 %) and declined to 28 % for the largest particles. Similarly, the H–C–C= carbon was the second most abundant WSOC type for particles with *δ*_p_ < 7.2 μm (25–34 %) and declined moderately to 17 % for the largest particles. The H–CO carbon accounted for approximately 49 % of the identified WSOC for particles with *δ*_p_ > 7.2 μm and decreased to 4 % of WSOC for particles with *δ*_p_ < 1.5 μm. The contribution of aromatic carbon to WSOC increased from 2 % for the smallest particles to 6 % for the larger particles, while acetalic carbon accounted for 1 % for all particle size ranges. The WSOC not associated with the five carbon types was negligible (less than 1 %) for particles with *δ*_p_ < 0.49 μm and increased to 47 % of WSOC for particles with 1.5 < *δ*_p_ < 3.0 μm and 22 % for larger particles. The carbon deficit may be related to carbon associated with carboxylic and/or hydroxyl groups and carbon atoms with no C–H bonds (e.g., quaternary C). Alkenoic acids and alcohols in urban environments have been shown to be accumulated in particles with 0.96 < *δ*_p_ < 3.0 μm (Kavouras and Stephanou, 2002). Overall, this analysis showed that aliphatic carbon originating from anthropogenic sources accounted for the largest fraction of fine and ultrafine WSOC. Sugars and other oxygenated compounds associated with biological particles dominated larger particles. Atmospheric aging appeared to be negligible during the monitoring period.

## 4 Conclusions

The functional characteristic of water soluble organic carbon for different particles sizes in an urban area during winter and spring has been studied. Using ^1^H-NMR fingerprints, ^13^C isotopic analysis and molecular tracers, the sources of particulate WSOC were reconciled for specific functional organic groups. A bimodal distribution was drawn for particle mass and water-soluble extract. WSOC and organic hydrogen were distributed between fine particles with MMADs of 0.37 and 0.34 μm and coarse particles with MMADs of 11.83 and 11.35 μm, indicating a mixture of primary large organic aerosol and condensed organic species in the accumulation mode. The NMR spectra for larger particles (*δ*_p_ > 3.0 μm) demonstrated a strong oxygenated saturated aliphatic content and the presence of fructose, sucrose, glucose, acetate, formate and succinate. These compounds have been previously found in pollen, soil and sea spray particles. For smaller particles (*δ*_p_ < 1.5 μm), the NMR spectra were dominated by saturated and unsaturated aliphatic protons. Organic species associated with biomass burning (i.e., levoglucosan) and urban traffic emissions (phthalate and terephthalate) were tentatively determined. Furthermore, resonances attributed to ammonium and amines were recognized, suggesting the presence of ammonium/aminium nitrate and sulfate secondary aerosol. The *δ*^13^C corroborated the local anthropogenic origin of fine and ultrafine organic aerosol. The values of the H–C–C=O / Σ (H–C–) and H–C–O / Σ (H–C–) ratios for the different particle sizes also confirmed the mixed contributions of urban and biomass burning emissions for fine and ultra-fine aerosol. The observed distribution of functional groups allowed for the distinct separation of biomass burning and pollen particles, in agreement with previous studies. More than 95 % of WSOC was associated with the five types of non-exchangeable organic hydrogen shown for the largest and smallest particle sizes. Overall, we characterized the WSOC in the southern Mississippi Valley, a region influenced by local anthropogenic sources, intense episodes of pollen, and regional secondary sources of anthropogenic and marine origin. We showed that NMR provides qualitative and, in conjunction with thermal optical reflectance and isotopic analysis, quantitative information on the compositional features of WSOC. Finally, the relative distribution of non-exchangeable organic hydrogen functional groups appeared to be distinctively unique for pollen particles and different than that previously observed for biomass burning and biogenic secondary organic aerosol, indicating that the origin of WSOC may be determined.

## Figures and Tables

**Table 1:** Major aerosol types and diagnostic ratios of PM_2.5_ chemical species in Little Rock, Arkansas during the study period.

Variable	Value (mean ± st. error)	Ratio	Value (mean ± st. error)
Ambient temperature (°C)	10.6 (6.4–16.6)	OE/EC	4.58 ± 1.06
Barometric pressure (torr)	758 (756–762)	Molar ${\text{NH}}_{4}^{+}/{\text{SO}}_{4}^{2-}$	3.07 ± 0.29
Organic mass (μg m^−3^)	5.5 ± 0.9	${\text{SO}}_{4}^{2-}/S$	2.66 ± 0.90
Elemental carbon (μg m^−3^)	0.7 ± 0.1	K^+^/K	1.00 ± 0.28
Ammonium sulfate and nitrate (μg m^−3^)	4.4 ± 1.6	K/Fe	0.87 ± 0.25
Soil dust (μg m^−3^)	0.5 ± 0.1	Ni/V	0.44 ± 0.41
Sea spray (μg m^−3^)	0.1 ± 0.1	Al/Si Al/Ca	0.40 ± 0.20 1.71 ± 0.82

**Table 2:** Particle mass, TWSE, WSOC and non-exchangeable organic hydrogen concentrations and *δ*^13^C at each impactor stage for urban aerosol.

	Diameter (μm)					
	30–7.2 μm	7.2–3.0 μm	3.0–1.5 μm	1.5–0.96 μm	0.96–0.49 μm	< 0.49 μm
Particle mass (μg m^−3^)	3.6 ± 0.8	3.5 ± 0.9	1.7 ± 0.3	1.6 ± 0.1	2.6 ± 0.1	11.2 ± 2.8
TWSE (μg m^−3^)	0.5 ± 0.1	1.0 ± 0.4	0.6 ± 0.2	0.7 ± 0.2	1.6 ± 0.1	5.4 ± 1.4
WSOC (μg m^−3^)	0.2 ± 0.1	0.1 ± 0.1	0.1 ± 0.1	0.1 ± 0.1	0.4 ± 0.1	1.2 ± 0.2
Total organic H (nmol m^−3^)	12.5 ± 0.9	7.8 ± 1.0	4.1 ± 0.1	5.7 ± 1.3	17.4 ± 3.5	73.9 ± 12.3
R–H (nmol m^−3^)	1.7 ± 0.3	1.9 ± 0.4	1.1 ± 0	2.6 ± 1.4	9.1 ± 2.5	33.8 ± 11.9
H–C–C= (nmol m^−3^)	1.4 ± 0.1	1.5 ± 0.1	0.9 ± 0.1	1.6 ± 0.8	6.4 ± 1.9	19.3 ± 8.4
H–C–O (nmol m^−3^)	9.0 ± 1.2	4.2 ± 1.6	1.9 ± 0.1	1.4 ± 0.2	1.7 ± 0.5	20 ± 2.7
O–CH–O (nmol m^−3^)	0.2 ± 0.2	0.1 ± 0.2	0.1 ± 0.1	0.1 ± 0.1	0.1 ± 0.1	0.5 ± 0.4
Ar–H (nmol m^−3^)	0.1 ± 0.1	0.1 ± 0.1	0.1 ± 0.1	0.1 ± 0.1	0.1 ± 0.1	0.3 ± 0.2
Molar H / C ratio	0.84 ± 0.02	0.92 ± 0.09	0.48 ± 0.02	0.48 ± 0.02	0.54 ± 0.05	0.73 ± 0.02
*δ*^13^C	−25.93 ± 0.31	−25.83 ± 0.19	−25.61 ± 0.05	−26.13 ± 0.11	−26.76 ± 0.22	−26.81 ± 0.18

**Table 3:** Mass median aerodynamic diameter (in μm) of particle mass, TWSE, WSOC and non-exchangeable organic hydrogen.

	Total	Coarse	Fine
Particle mass	0.68 ± 0.19	9.15 ± 2.75	0.39 ± 0.03
TWSE	0.46 ± 0.02	6.35 ± 0.45	0.39 ± 0.02
WSOC	0.43 ± 0.02	11.83 ± 2.20	0.37 ± 0.01
Organic hydrogen	0.41 ± 0.01	11.35 ± 1.45	0.34 ± 0.01
R–H	0.37 ± 0.01	7.00 ± 0.01	0.34 ± 0.01
H–C–C=	0.41 ± 0.03	7.13 ± 0.03	0.37 ± 0.02
O–C–H	0.48 ± 0.02	13.05 ± 1.95	0.31 ± 0.01
O–CH–O	0.73 ± 0.07	10.25 ± 0.25	0.40 ± 0.04
Ar–H	1.25 ± 0.65	10.10 ± 0.90	0.53 ± 0.12
